# Time-Resolved Study of Polaron-Mediated Relaxation in Ferromagnetic La_0.67_Sr_0.33_MnO_3_/BiFeO_3_ Thin Films by Dual-Color Pump–Probe Spectroscopy

**DOI:** 10.1021/acsomega.6c02859

**Published:** 2026-06-24

**Authors:** Le Thi Cam Tuyen, Chi-Yen Huang, Shin Rou Yin, Nguyen Nhat Quyen, Chih-Wei Luo, Yen-Lin Huang

**Affiliations:** † Department of Materials Science and Engineering, 34914 National Yang Ming Chiao Tung University, Hsinchu 30010, Taiwan; ‡ Department of Chemical Engineering and Biotechnology, College of Engineering, Tatung University, Taipei 10452, Taiwan; § Department of Electrophysics, National Yang Ming Chiao Tung University, Hsinchu 30010, Taiwan; ∥ National Synchrotron Radiation Research Center, Hsinchu 30010, Taiwan; ⊥ Taiwan Consortium of Emergent Crystalline Materials (TCECM), National Science and Technology Council, Taipei 10601, Taiwan; # Institute of Physics and Center for Emergent Functional Matter Science, National Yang Ming Chiao Tung University, Hsinchu 30010, Taiwan; ∇ Department of Physics, University of Washington, Seattle, Washington 98195, United States; ○ Center for Emergent Functional Matter Science, National Yang Ming Chiao Tung University, Hsinchu 30010, Taiwan

## Abstract

La_0.67_Sr_0.33_MnO_3_/BiFeO_3_ (LSMO/BFO) ferromagnet/antiferromagnet heterostructure thin films were grown on SrTiO_3_ (100) substrates by pulsed-laser deposition, and their strain-dependent magnetic properties and ultrafast relaxation dynamics were investigated in comparison with single-layer LSMO films. For LSMO/BFO bilayers, increasing the BFO thickness from 40 to 120 nm reduces the coercive field from 8.34 to 3.52 mT and lowers the Curie temperature from 343 to 295 K, consistent with a BFO-thickness–controlled strain state in the LSMO layer that suppresses magnetization. In contrast, single-layer LSMO films exhibit increasing coercive field and Curie temperature with increasing LSMO thickness. Dual-color pump–probe spectroscopy further reveals a marked acceleration of carrier–lattice relaxation in LSMO/BFO: the electron–lattice coupling time decreases from 10.07 to 0.65 ps as BFO thickness increases, accompanied by GHz-range strain-pulse oscillations (18.99–24.48 GHz) and spin–lattice relaxation times approaching ∼100 ps. Single-layer LSMO shows much slower electron–lattice relaxation (>200 ps) and higher-frequency oscillations spanning ∼107.84–229.46 GHz, as determined from both FFT analysis and fitting of the oscillatory component, consistent with small-polaron-dominated transport. Together, these results demonstrate that BFO thickness provides an effective handle to tune strain, magnetic response, and ultrafast carrier–lattice dynamics in LSMO-based heterostructures, with implications for energy-conversion and spintronic applications through improved carrier transport efficiency and tunable magnetic functionalities.

## Graphical abstract

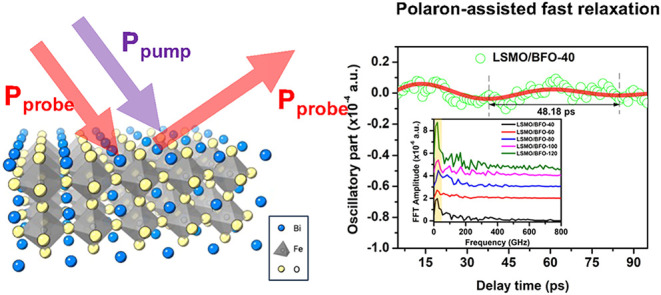

## 1 Introduction

Perovskite-based thin-film heterostructures have emerged as a cornerstone in condensed matter physics, captivating researchers with their extraordinary tunability across the electronic, magnetic, and optical domains. These materials offer a remarkable platform for understanding fundamental physics while paving the way for transformative technologies in energy conversion, spintronics, and multifunctional devices.^1–5^ Among the many systems explored, heterostructures such as La_0.67_Sr_0.33_MnO_3_ (LSMO) and BiFeO_3_ (BFO) stand out for their unique combinations of ferromagnetic, antiferromagnetic, and ferroelectric properties. This coexistence of order parameters fosters the emergence of complex phenomena, including Jahn–Teller polarons, spin–lattice coupling, and polaron–phonon interactions, making these systems pivotal in advancing material functionality.^6–8^ Their diverse properties increase their scientific significance, making them attractive contenders for future advanced devices.

Central to the innovation in these systems is the dynamic strain environment at the interface of LSMO and BFO layers. The mismatch in lattice parameters and mechanisms of strain relaxation play a crucial role in determining their fundamental properties. More importantly, these factors modulate interfacial electronic and magnetic behaviors, creating a fertile ground for research into emergent phenomena.^9,10^ For instance, BFO’s multiferroic nature—characterized by its coexistence of ferroelectricity and antiferromagnetism—introduces a layer of complexity that enables strain-mediated modulation of LSMO’s magnetic properties. This has led to advancements such as electric field-controlled exchange bias in LSMO/rhombohedral BFO heterostructures, a development that could pave the way for compact, energy-efficient devices.^11^ These interactions critically affect magnetic parameters such as coercivity, Curie temperature, and magnetization. Additionally, charge transport and lattice dynamics in systems such as LSMO/BFO and La_0.7_Ca_0.3_MnO_3_/BFO heterostructures are often influenced by mechanisms such as magnon–polaron coupling, which is central to tailoring their multifunctional capabilities.^12,13^ These findings underscore the necessity for meticulous control over growth techniques and structural configurations in designing LSMO/BFO heterostructures. Recent studies further highlight the role of interfacial strain and exchange coupling in driving these complex interactions within oxide heterostructures.^14,15^

Exploring such phenomena requires advanced experimental techniques capable of probing nanoscale and time-resolved dynamics. Among these, time-resolved pump–probe spectroscopy has proven to be particularly effective for investigating electronic and lattice interactions in perovskite materials.^16,17^ For instance, Guzelturk and co-workers utilized femtosecond-resolution diffuse X-ray scattering to conduct momentum-resolved phonon spectroscopy, revealing nanoscale structural distortions and radially expanding strain fields associated with polaron formation and relaxation on picosecond time scales in photoexcited lead halide perovskites, specifically (MA)­PbBr_3_.^17^ Similarly, Cinquanta et al. employed ultrafast optical pump–THz probe spectroscopy combined with DFT calculations to uncover the role of large polarons in the dielectric response of CsPbBr_3_ nanocrystal thin films.^16^ Thus, the time-resolved pump–probe technique enables precise observation of relaxation dynamics and differentiation between small and large-polaron formation. By capturing the interplay between electronic and lattice degrees of freedom, it provides critical insights into how polarons influence charge transport and magnetic behaviors, paving the way for advancements in LSMO/BFO-based devices such as energy-efficient electronics and spintronics. For example, this technique has revealed a coupling between small polarons in ultrathin LSMO films and acoustic phonons in STO substrates, emphasizing their potential for manipulation in device applications involving colossal magnetoresistance materials.^7^ Similarly, ultrafast optical spectroscopy studies by Sheu et al. demonstrated that tunable magnetotransport in a related compound, La_0.7_Ca_0.3_MnO_3_/BFO heterostructures, arises from polaronic behavior linked to interfacial antiferromagnetic order. These investigations revealed a dynamic spectral weight transfer dependent on ferroelectric polarity, magnetic fields, and spin alignment at low temperatures, providing critical insights into magnetoelectric coupling mechanisms and multiferroic functionalities.^13^ The ability to probe such ultrafast phenomena highlights the importance of precise experimental control, enabling deeper understanding and manipulation of material properties. Collectively, these approaches pave the way for the development of advanced materials and technologies with enhanced energy-conversion efficiency, optimized charge transport, and improved magnetic tunability—marking a significant step forward in materials science and engineering.

In this study, we prepared LSMO/BFO heterostructures synthesized via pulsed-laser deposition (PLD). Our research focuses on the influence of the BFO layer thickness in modulating strain and interface-driven phenomena, providing a systematic analysis of how these factors impact polaron dynamics and magnetic behavior. We demonstrate that structural strain, tuned by the BFO layer, induces a transition from small to large polarons in the LSMO thin films. This transition is accompanied by shorter electron–lattice coupling times and reduced coercivity, indicating that the BFO layer effectively mitigates the strain and enhances polaron mobility. These findings establish a clear connection between structural parameters and functional properties, shedding light on the underlying physical mechanisms. By systematically elucidating the role of BFO thickness, our study lays the foundation for leveraging LSMO/BFO heterostructures in multifunctional applications, emphasizing their potential for advancing both fundamental research and technological innovation.

## 2 Experimental Section

The ferromagnet/antiferromagnet LSMO/BFO heterostructures were fabricated on SrTiO_3_ (STO (100)) single-crystal substrates using PLD. The BFO layer were deposited onto STO substrates with thicknesses ranging from 40 to 120 nm, controlled by varying the number of laser pulses with a 5 Hz repeat rate and laser fluence of 1.5 J/cm^2^. The BFO layers were deposited at 700 °C under an oxygen pressure of 1.5 × 10^–1^ Torr of 1.5 × 10^–1^ Torr. Subsequently, a 120 nm thick LSMO layer was grown atop the BFO layer at 750 °C under an oxygen pressure of 2.0 × 10^–1^ Torr. We also prepared the single layer of LSMO as the controlled sample with the same growth parameters as the LSMO in the heterostructure.

The crystal structures were characterized using X-ray diffraction (XRD, D2-Phaser, Bruker, Germany) with Cu Kα_1_ radiation (λ = 1.540598 Å). X-ray reflectivity (XRR) measurements were performed to determine the films’ thicknesses at the 09A and 17B beamlines of the National Synchrotron Radiation Research Center (NSRRC) in Taiwan, with data analysis conducted using the GenX reflectivity software. The surface morphology of the films was examined using an atomic force microscope (AFM, Bruker on Minus K) in contact mode over 5 μm × 5 μm areas. The AFM data were subsequently analyzed using Gwyddion 2.63 software. Magnetic properties were measured using a vibrating sample magnetometer (VSM, Versa Lab, Quantum Design) across a temperature range of 50–380 K.

To investigate polaron–phonon coupling in the LSMO/BFO heterostructures, dual-color pump–probe spectroscopy was employed.^18^ A schematic diagram of the pump–probe system is shown in Figure 1. The system utilizes the output of a femtosecond Ti laser (800 nm, 100 fs pulse duration, 5.2 MHz repetition rate), which is split into pump and probe beams by a beam splitter (BS). The pump beam is directed through a β-BaB_2_O_4_ (BBO) nonlinear crystal to convert the wavelength from 800 to 400 nm, following modulation by an acoustic-optic modulator. The pump beam’s reference frequency is controlled by a lock-in amplifier. The beam is then passed through a delay stage to adjust its optical path, with polarizers (P) converting normal light to polarized light. A half-wave plate (HWP) is placed in the pump’s optical path to align the polarization planes of the pump and probe beams at a 90-degree angle. The pump beam is focused onto the sample to excite it into nonequilibrium states, while the probe beam measures changes in the carrier dynamics of the thin-film material. The fluences of the pump and probe beams were 45.7 μJ/cm^2^ and 3.5 μJ/cm^2^, respectively. Transient reflectivity changes (Δ*R*) were detected using a photoreceptor–lock-in amplifier system, with the time delay between the pump and probe pulses being systematically controlled.

**Figure 1:**
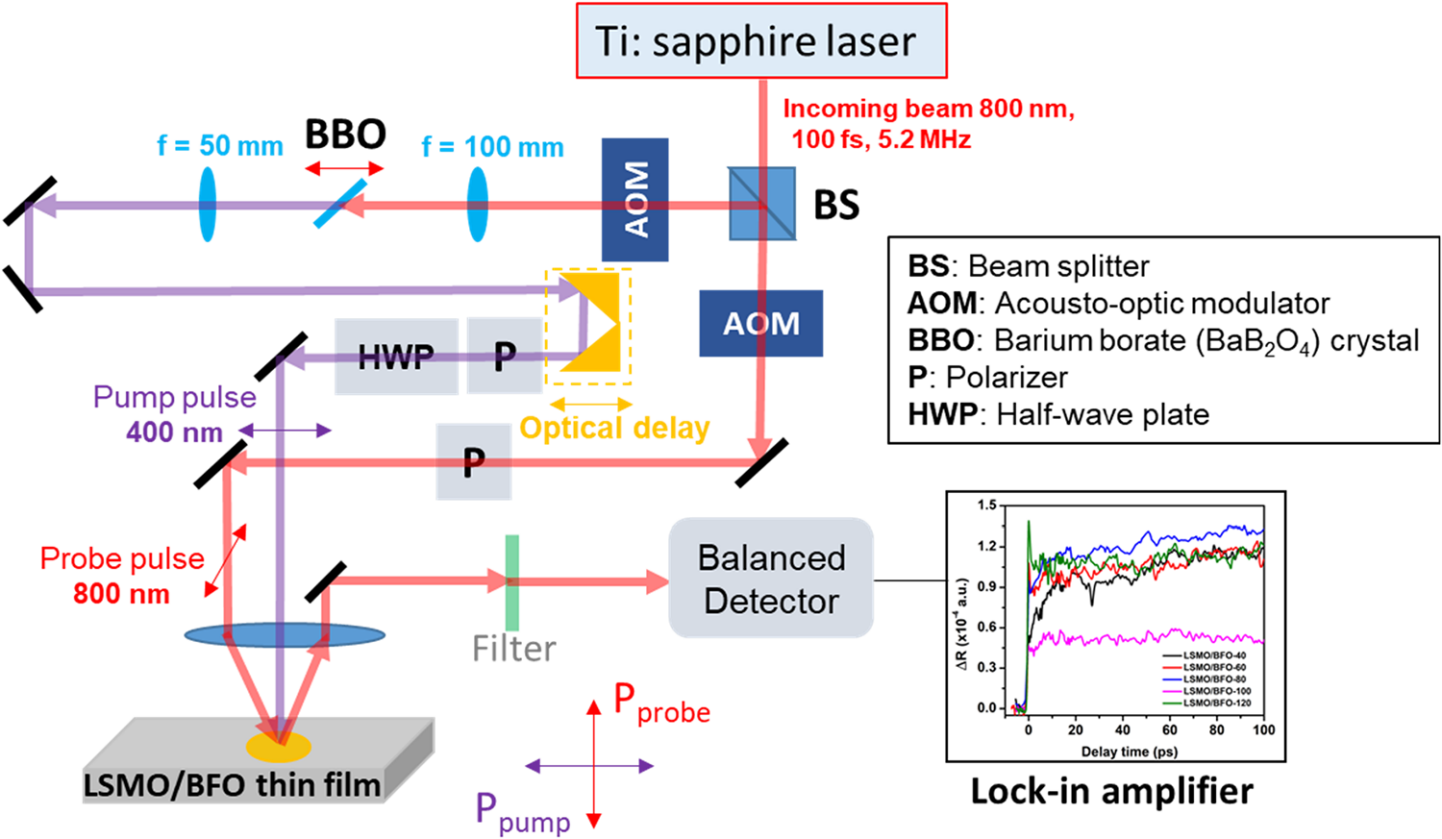
A dual-color pump–probe setup was used for the transient reflectivity experiments.

## 3 Results and Discussion

### 3.1 Structural Analysis

Figure 2 presents the XRD and XRR measurement results for the LSMO/BFO and LSMO thin films. Figure 2a shows the XRD patterns of LSMO/BFO thin films on STO (001) substrates with BFO thicknesses (*t*_BFO_) of 40, 60, 80, 100, and 120 nm, labeled as LSMO/BFO-40, LSMO/BFO-60, LSMO/BFO-80, LSMO/BFO-100, and LSMO/BFO-120, respectively. The BFO layers exhibit a (00*l*) preferred orientation characteristic of the rhombohedral phase, aligning closely with the standard XRD pattern for bulk BFO (JCPDS #86-1518). However, the (001) Bragg peak shifts slightly to a lower angle compared to the bulk BFO peak at 45.73° (see the inset of Figure 2a), corresponding to an increased *c*-axis lattice constant of approximately 3.979 Å, which is higher than the bulk value of 3.965 Å. This shift suggests an out-of-plane tensile strain in the BFO thin films due to the lattice mismatch with the STO substrate. The LSMO layer exhibits a pure (00*l*) orientation, with a *c*-axis lattice constant of approximately 3.876 Å, slightly larger than the bulk pseudocubic value of 3.873 Å. This indicates that the LSMO layer experiences in-plane compressive strain due to lattice mismatch with the BFO layer.

**Figure 2:**
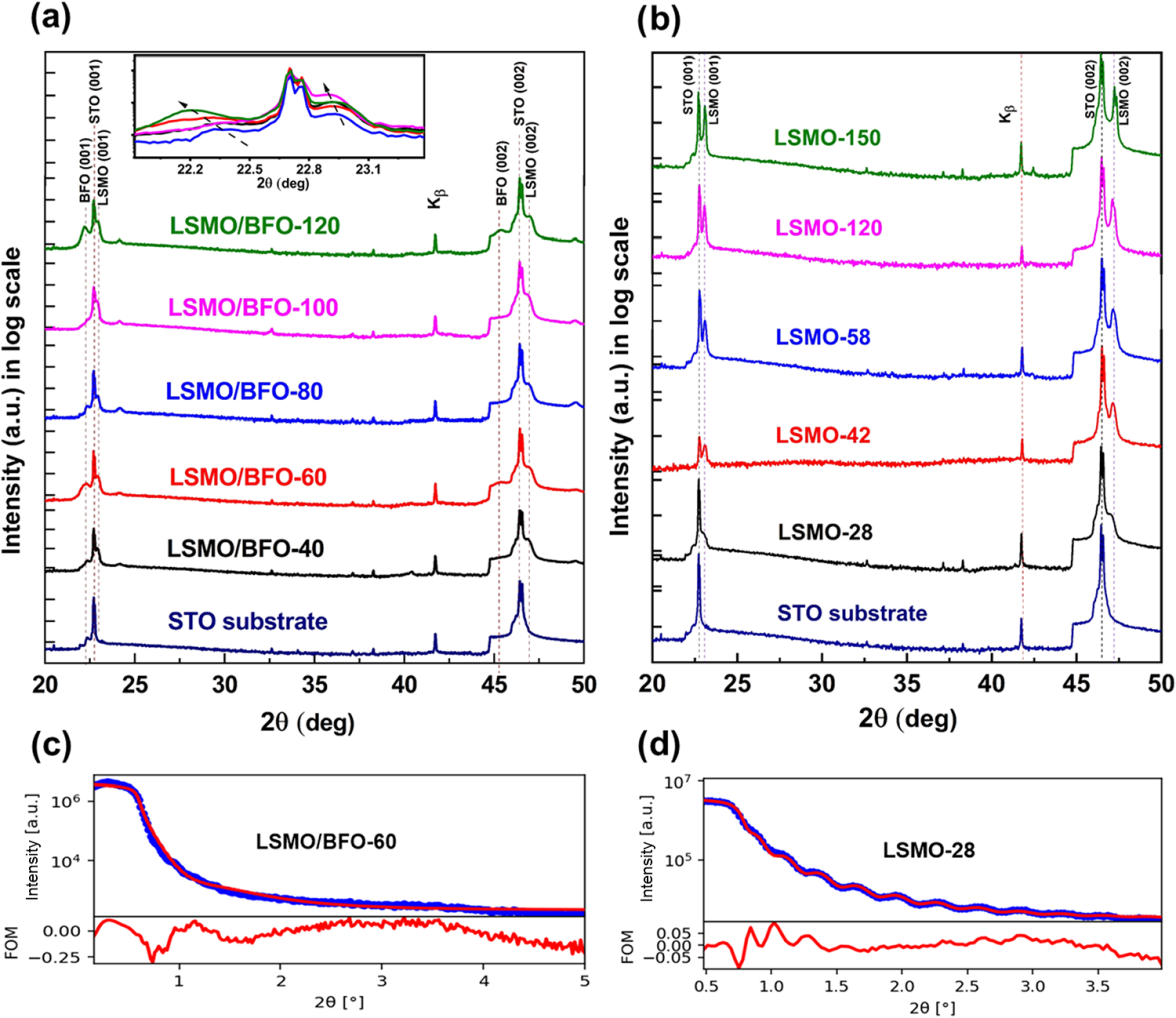
(a) XRD patterns of bilayer LSMO/BFO thin films, where LSMO/BFO-40 = LSMO (120 nm)/BFO (40 nm), LSMO/BFO-60 = LSMO (120 nm)/BFO (60 nm), LSMO/BFO-80 = LSMO (120 nm)/BFO (80 nm), LSMO/BFO-100 = LSMO (120 nm)/BFO (100 nm), and LSMO/BFO-120 = LSMO (120 nm)/BFO (120 nm). (b) XRD patterns of LSMO thin films with varying LSMO thicknesses of 28, 42, 58, 120, and 150 nm, labeled as LSMO-28, LSMO-42, LSMO-58, LSMO-120, and LSMO-150, respectively. (c, d) XRR measurements of LSMO/BFO-40 and LSMO-28 thin films, fitted using GenX 3.6.22 software.

Figure 2b displays the XRD patterns for LSMO thin films on STO (001) substrates with LSMO thicknesses (*t*_LSMO_) of 28, 42, 58, 120, and 150 nm, labeled as LSMO-28, LSMO-42, LSMO-58, LSMO-120, and LSMO-150, respectively. All LSMO thin films display a pronounced (00*l*) preferred orientation, with an in-plane lattice parameter measured at 3.855 Å. This value, being slightly smaller than the bulk lattice parameter, indicates the presence of in-plane compressive strain within the LSMO layer. In our study, the lattice mismatch between LSMO and STO is approximately 1.28%, smaller than the mismatch between LSMO and BFO (∼2.61%). This larger lattice mismatch in the LSMO/BFO system results in broader and less distinct XRD peaks in the LSMO layers grown on BFO, compared to those grown directly on the STO substrate (see Figure 2a). The peak broadening observed is attributed to strain resulting from the lattice mismatch between the thin-film layers and the substrate.

Figure 2c,d shows the XRR results of the LSMO/BFO and the single LSMO layer. For the LSMO/BFO-60 sample (Figure 2c), the fitted curve obtained using GenX 3.6.22 software^19^ indicates that the LSMO layer has a thickness of approximately 120 nm, while the BFO layer is around 60 nm thick. Similarly, the single-layer LSMO-28 sample (Figure 2d) has a thickness of approximately 28 nm, as determined from the fitting analysis. The XRR data for other LSMO/BFO bilayer samples consistently reveal an LSMO layer thickness of 120 nm with varying BFO layer thicknesses of 60, 80, 100, and 120 nm. In the case of single-layer LSMO films, the thicknesses increase progressively with values of 42, 58, 120, and 150 nm. These findings highlight the precise thickness control achievable with XRR analysis, offering valuable structural information that is essential for optimizing these thin films for potential applications in electronics and spintronics.

### 3.2 Magnetic Properties and Jahn–Teller Polarons Observation

Figure 3a,b shows the magnetization behavior of the LSMO/BFO heterostructure under an applied magnetic field parallel to the film surface (in-plane direction), along with the temperature dependence of the magnetization. The magnetization curves of the LSMO/BFO heterostructures reveal a significant decrease in the coercive field (*H*_C_) from 8.34 to 3.52 mT as the BFO layer thickness increases from 40 to 120 nm. This reduction is attributed to changes in the in-plane lattice strain of the LSMO layer induced by the underlying BFO layer. As *t*_BFO_ increases, the LSMO layer experiences greater tensile strain due to an enhanced lattice mismatch, which weakens the exchange interactions and decreases magnetization. The magnetic saturation of the LSMO/BFO thin-film ranges from 50 to 250 emu/cm^3^, which is comparable to the values reported in the literature.^20^

**Figure 3:**
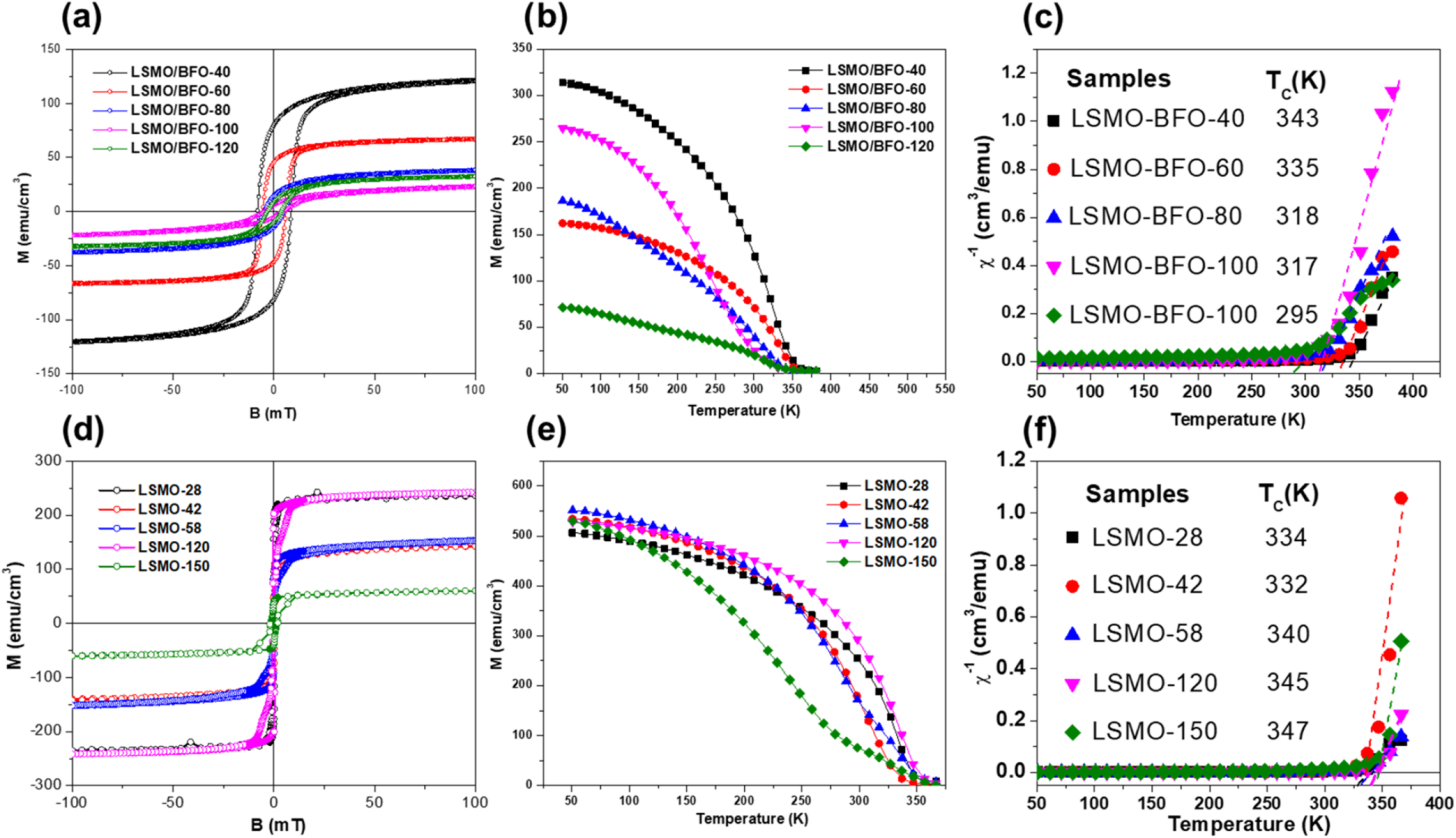
(a, d) Magnetization-field and temperature-dependent magnetization curves for LSMO/BFO heterostructure thin films. (b, e) Magnetization-field and temperature-dependent magnetization curves for LSMO thin films. (c, f) Temperature dependence of inverse magnetic susceptibility for LSMO/BFO heterostructures with varying BFO thicknesses and LSMO thin films, measured at 100 mT. The dashed line corresponds to the Curie–Weiss fitting applied to the inverse susceptibility curve within the high-temperature paramagnetic region.

A similar trend is observed in the Curie temperature (*T*_C_), which decreases from 343 to 295 K with increasing *t*_BFO_, as shown in Figure 3c. This behavior is consistent with the Jahn–Teller effect, which describes how increased tensile strain in the LSMO lattice reduces the orbital overlap between manganese ions and oxygen ions, thereby lowering *T*_C_.^9,21^ The strain effects are corroborated by structural changes observed in the thin films in the XRD results. Indeed, the mismatch value between the LSMO and BFO layers increases from 2.34% to 2.85% as the thickness of the BFO layer increases from 40 to 120 nm. The mismatch between the films and the substrate, or between individual layers, plays a critical role in modulating the tensile strain within the thin films. The Curie–Weiss law, χ = *C*/(*T* – *T*_C_), was used to determine the magnetic transition temperature, where χ represents magnetic susceptibility, T is the absolute temperature, *T*_C_ is the Curie temperature, and *C* is the Curie constant. Figure 3c displays the inverse magnetic susceptibility (1/χ) curves as a function of temperature for all four samples, illustrating the observed trends in *T*_C_. The observed reductions in *H*_C_ and *T*_C_ align well with the tensile strain model, highlighting the critical role of strain engineering in tailoring the magnetic properties of LSMO/BFO bilayers.

Figure 3d shows that both the coercive field and the Curie temperature increase with the thickness of the LSMO film. Interestingly, the *H*_C_ and *T*_C_ of the 28 nm LSMO film are higher than those of the 42 nm sample. This phenomenon can be explained by the stronger pinning of magnetic domains to the substrate in thinner films. The increased strain resulting from this strong pinning in thinner films leads to higher *H*_C_ and *T*_C_ values.^22^ The magnetic properties of LSMO thin films are significantly influenced by the presence of a BFO layer due to interfacial coupling effects. While stand-alone LSMO-120 films exhibit robust ferromagnetic behavior with high saturation magnetization (∼235 emu cm^–3^) and a *T*_C_ of 345 K, the inclusion of a BFO layer introduces interaction between Fe^3+^ and Mn^3+/4+^ and reduces the saturation magnetization due to spin frustration at the interface (see Figure 3a).^23^ Additionally, tensile strain from BFO alters the magnetic anisotropy and slightly lowers the *T*_C_.^9^ These interfacial interactions make LSMO/BFO heterostructures promising for strain-engineered spintronic applications.

In the time-resolved pump–probe spectroscopy experiments, the pump and probe beam fluences were set to 45.7 and 3.5 μJ/cm^2^, respectively, to investigate Jahn–Teller polarons in LSMO/BFO thin films. The transient reflectivity change (Δ*R*) of LSMO/BFO thin films with varying BFO thickness was measured at room temperature and is presented in Figure 4a. The results reveal strain-pulse oscillations, albeit with significant noise. Magnetoelectric coupling in LSMO/BFO heterostructures, driven by ferroelectric polarization at the interface, modulates magnetization and exchange bias,^9^ directly influencing the optical and electronic signals observed during pump–probe experiments.^24^ We note that the oscillatory backgrounds and reduced signal-to-noise may arise from acoustic strain pulses, surface roughness/scattering, and thickness-dependent interference effects.

**Figure 4:**
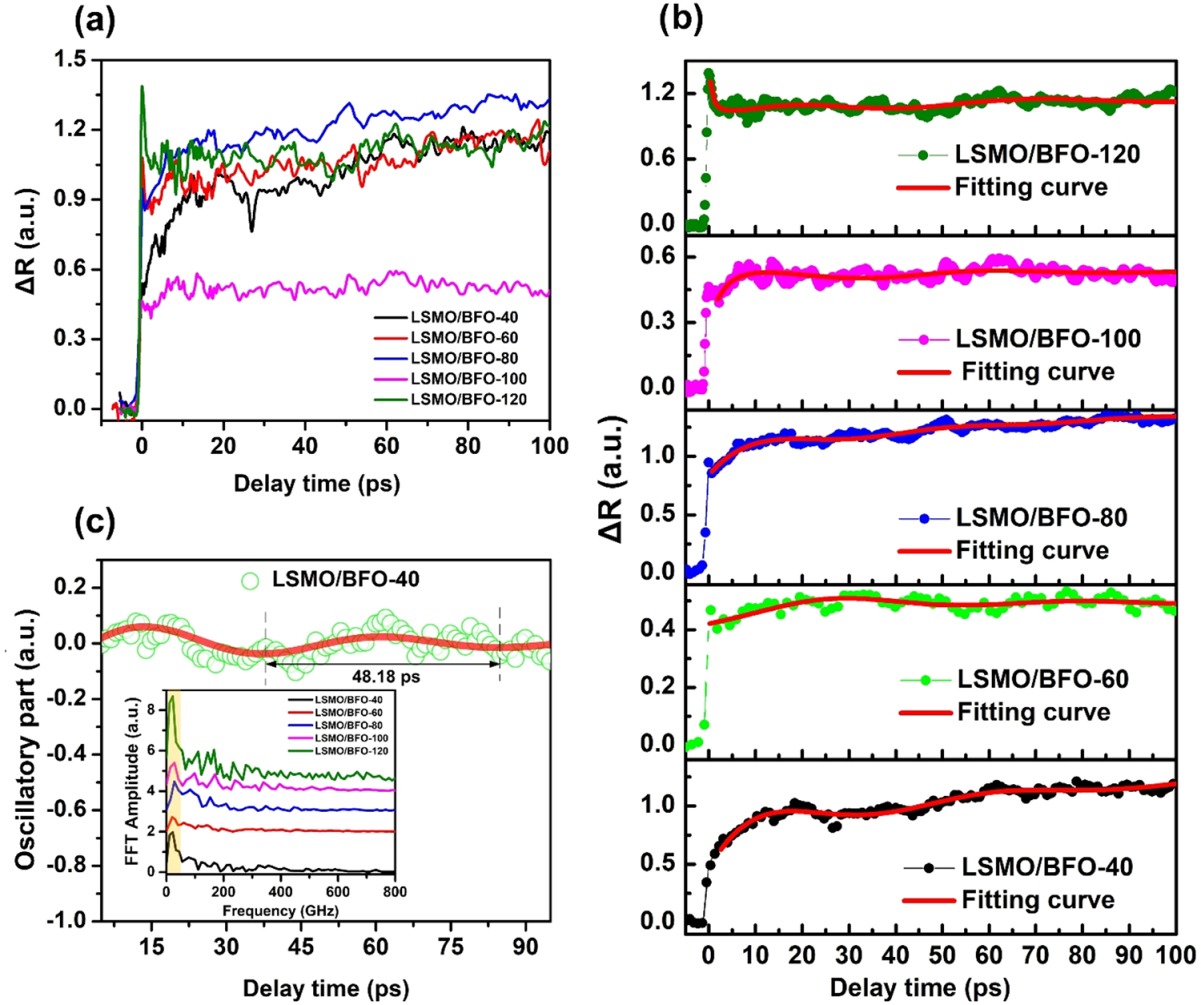
(a) Transient changing reflectivity (Δ*R*) of LSMO/BFO thin films with different t_BFO_ measured at room temperature. (b) Transient changing reflectivity signal of the LSMO/BFO bilayer together with the exponential fitting curve by using eq 1. (c) Oscillatory component of the LSMO/BFO-40 sample and Fast Fourier Transform (FFT) of the oscillation part of LSMO/BFO thin films.

Previous research using static spectroscopy on LSMO has shown that the 3.1 eV pump beam photoenergy surpasses the bandgaps of LSMO (2 eV) and BFO (2.7 eV).^25,26^ When the pump beam irradiates the material, hot electrons are excited from the valence band to the upper conduction band, leading to a rapid increase in reflectivity. As depicted in Figure 4a, the transient reflectivity of the probe beam shows a sharp initial decrease within 5 fs, followed by a gradual relaxation to a plateau over hundreds of picoseconds. The hot carrier relaxation process in LSMO/BFO thin films can be divided into two main components: (1) a fast component associated with electron–lattice coupling and (2) a slower component linked to spin–lattice coupling. To analyze the electron dynamics, eq 1, which includes an exponential decay function, was employed.^26^

1 $$\frac{\Delta R}{R}=A_{el‐la}\exp\exp\left(-\frac{t}{T_{el‐la}}\right)+A_{sp‐la}\exp[1-\exp\left(-\frac{t}{T_{sp‐la}}\right)]\exp\left(-\frac{t}{T_{mag}}\right){+A}_{osc}\cos\left(\omega t+\varphi \right)\exp\exp\left(\frac{-t}{T_{osc}}\right)+y_{0}$$

where *A*_el‑la_ and *T*_el‑la_ are the amplitude and time of electron–lattice relaxation, respectively; *A*_sp‑la_ and *T*_sp‑la_ are the amplitude and time of spin–lattice relaxation, respectively; and *T*_mag_ is the time of magnetic order recovery. *A*_osc_, ω, φ, and *T*_osc_ are the intensity, frequency, phase, and amplitude decay time of strain-pulse oscillation, respectively, and y_0_ is the amplitude of the background.

Figure 4b shows the time-resolved pump–probe spectroscopy fitting results of LSMO on BFO thin films with different BFO thicknesses. Fast component due to the electron–lattice coupling, *T*_el‑la_ decreases from 10.07 to 0.65 ps with increasing BFO layer thickness (as shown in Table 1). For the ferromagnetic metallic state, T_el‑la_ is found to be smaller than 100 ps, indicating the existence of large polarons.^27–29^ Therefore, *T*_el‑la_ is decreased by almost an order of magnitude due to the formation of larger polarons, which are known to enhance carrier transport by reducing electron–phonon scattering and promoting carrier delocalization.^30^ While the reduction in *T*_el‑la_ with increasing BFO thickness is primarily attributed to large-polaron formation and enhanced polaron–phonon coupling at the LSMO/BFO interface, consistent with reports of interfacial ion diffusion modifying local carrier dynamics in perovskite systems,^31^ interface-induced defects and strain inhomogeneity may also contribute to the relaxation dynamics. The relatively large lattice mismatch (∼2.61%) can introduce structural defects and local strain variations near the interface, which may act as additional scattering channels for photoexcited carriers. Furthermore, as discussed in the Section 3.1 (Figure 2a), XRD peak broadening suggests increasing strain inhomogeneity, potentially leading to a distribution of local lattice environments and relaxation times. Nevertheless, the systematic evolution of *T*_el‑la_ and its correlation with oscillation frequency support the dominant role of strain-mediated polaron dynamics in the present system.

**Table 1:** Dual Pump–Probe Fitting and FFT-Derived Parameters of LSMO/BFO Thin Films^a^

	fitting results (eq 1)						FFT results	
samples	*T*_el‑la_ (ps)	*T*_sp‑la_ (ps)	*T*_mag_ (ps)	*T*_osc_(ps)	ω (rad/ps)	*f* ^ω^ (GHz)	*f* ^FFT^ (GHz)	*T*_osc_^FFT^ (ps)
LSMO/BFO-40	10.07	90.42	36.61	52.33	0.12	18.99	19.93	50.18
LSMO/BFO-60	9.97	88.54	34.60	49.17	0.12	19.46	20.24	49.41
LSMO/BFO-80	8.33	84.33	29.50	47.31	0.15	24.47	26.75	37.38
LSMO/BFO-100	2.99	76.86	14.42	40.56	0.12	19.81	27.10	36.90
LSMO/BFO-120	0.65	69.28	10.96	38.36	0.14	22.77	22.18	45.09

a The oscillation period obtained from FFT (*T*_osc_^FFT^ = 1/*f* ^FFT^) is provided alongside the fitting results for comparison.

Slow component contributed by the spin–lattice coupling, *T*_sp‑la_ larger than *T*_el‑la_ and increases from 69.28 ps to 90.42 ps when the BFO layers are thicker. The time of magnetic order recovery (*T*_mag_) shows a decreasing trend when the *t*_BFO_ increases due to the magnetic properties being weaker for the thicker films. The amplitude decay time of strain-pulse oscillation (*T*_osc_) (due to the polaron–phonon oscillation) decreases caused by the smaller magnetization in the thicker films. The Jahn–Teller polaron–phonon coupling in LSMO leads to significant localization of the e_g_ electrons, which leads to smaller magnetization.^6^ Noticeably, the pump–probe results agree with the magnetic properties measurement. In addition, to confirm that LSMO/BFO thin films exhibit the larger polaron, the frequency of strain-pulse oscillation (*f*) was analyzed by using the Fast Fourier Transform (FFT) (see the inset of Figure 4c) and the value of ω (ω observed from the fitting of the pump–probe signal). The FFT results reveal an increasing frequency trend, ranging from 19.93 to 27.10 GHz (*f*:FFT, as shown in Table 1), suggesting the presence of large polarons in the LSMO/BFO thin films.^29^ Similarly, the frequencies calculated using ω, also presented in Table 1, show an increase from 18.99 to 24.48 GHz, albeit slightly lower than those estimated via FFT analysis. This consistency further supports the likelihood of large polarons being observed in the LSMO/BFO thin films. The decay time of the strain-pulse oscillation component was determined and analyzed. It should be noted that the GHz-range oscillations observed in the transient reflectivity of LSMO/BFO heterostructures may arise from multiple contributions, including both polaron–phonon coupling and coherent acoustic phonons (CAPs). CAPs can be generated by ultrafast thermoelastic excitation and propagate as strain pulses, giving rise to oscillatory reflectivity signals in pump–probe measurements.^32,33^ In multilayer structures such as LSMO/BFO, acoustic reflections at interfaces may further modulate these oscillations. In addition, thickness-dependent optical interference effects may also influence the measured transient reflectivity signal. Nevertheless, the systematic evolution of oscillation frequency with BFO thickness, together with its correlation with the reduction in Tel-la, supports the interpretation that polaron–phonon coupling remains the dominant contribution in the present system. Figure 4c presents the oscillation component extracted from the pump–probe signal by subtracting its nonoscillatory part for the LSMO/BFO-40 sample. The oscillation period is determined from the dominant FFT peak (∼19.93 GHz), corresponding to a period of approximately 50.18 ps. This value is consistent with the fitting result obtained using eq 1 (*T*_osc_ = 52.33 ps, Table 1), confirming the reliability of the analysis.

Table 1 presents key trends in the electron–lattice, spin–lattice, and strain-pulse oscillation dynamics of LSMO/BFO heterostructures as increasing BFO layer thickness. With increasing BFO thickness, the electron–lattice relaxation time *T*_e‑l_ significantly decreases from 10.07 ps (40 nm) to 0.65 ps (120 nm), consistent with the manifestation of large-polaron behavior, which enhanced electron–lattice coupling.^27^ This dynamic is supported by studies showing that large polarons exhibit faster relaxation times due to their delocalized interaction with the lattice.^28^ Similarly, the spin–lattice relaxation time *T*_sp_ decreases from 90.42 ps (40 nm) to 69.28 ps (120 nm), indicating a moderate enhancement in spin–lattice coupling. The magnetic order recovery time *T*_mag_ also sharply reduces from 36.61 ps (40 nm) to 10.96 ps (120 nm), suggesting that thicker films experience weaker magnetic properties, likely due to the dominance of polaron-mediated dynamics over magnetic ordering. Additionally, the strain-pulse oscillation amplitude decay time (*T*_osc_) decreases with increasing BFO thickness, indicating faster dissipation of strain energy. This trend aligns with findings from David Emin’s study, which demonstrated that the coupling between polarons and strain accelerates relaxation dynamics.^34^ The oscillation frequency, derived from both ω and Fast Fourier Transform (FFT) analyses, falls within the range of tens of GHz. This consistency between the two methods validates the accuracy and reliability of using eq 1 as a fitting model for the pump–probe signal analysis. These observations provide a new understanding of types of polaron in ferromagnet/antiferromagnetic heterostructured LSMO/BFO thin films and are crucial to understanding the charge transport in perovskites materials related to energy-conversion applications.

Figure 5a–e illustrates the transient reflectivity changes (Δ*R*), corresponding fitting curves, and oscillatory components for LSMO thin films with thicknesses of 28, 42, 58, 120, and 150 nm. Each panel depicts the evolution of Δ*R* as a function of delay time for a specific thickness, alongside the fitting results and extracted oscillatory components from the transient signals. The insets provide the FFT spectra of the oscillatory components, highlighting the dominant frequencies. These analyses leverage pump–probe measurements to investigate the role of polarons in LSMO thin films. The first component of the reflectivity dynamics, characterized by a relaxation time (*T*_el–la_) exceeding 200 ps, is attributed to strong electron–lattice coupling and the presence of Jahn–Teller small polarons.^35^ This is supported by an enhancement of *T*_el–la_ by nearly an order of magnitude, consistent with findings reported by Ren et al.,^29^ which confirm the formation of Jahn–Teller polarons in similar systems. The second component, with a spin–lattice relaxation time (*T*_sp–la_) below 20 ps, arises from spin–lattice coupling. Notably, *T*_sp–la_ increases with LSMO layer thickness, underscoring the impact of thickness on spin–lattice interactions. Additionally, the magnetic order recovery time (*T*_mag_) demonstrates a similar thickness-dependent trend, increasing with film thickness. This observation suggests that thicker films exhibit stronger magnetic properties, consistent with the magnetic analysis discussed earlier. These results underscore the dominant role of small polarons in the dynamical processes of LSMO thin films and align closely with prior studies.^7,29,34^ The observed oscillation period (*T*_osc_) from the extracted oscillation component is lower than 10 ps with increasing *t*_LSMO_. This closely aligns with the *T*_osc_ values derived from eq 1, as presented in Table 2.

**Figure 5:**
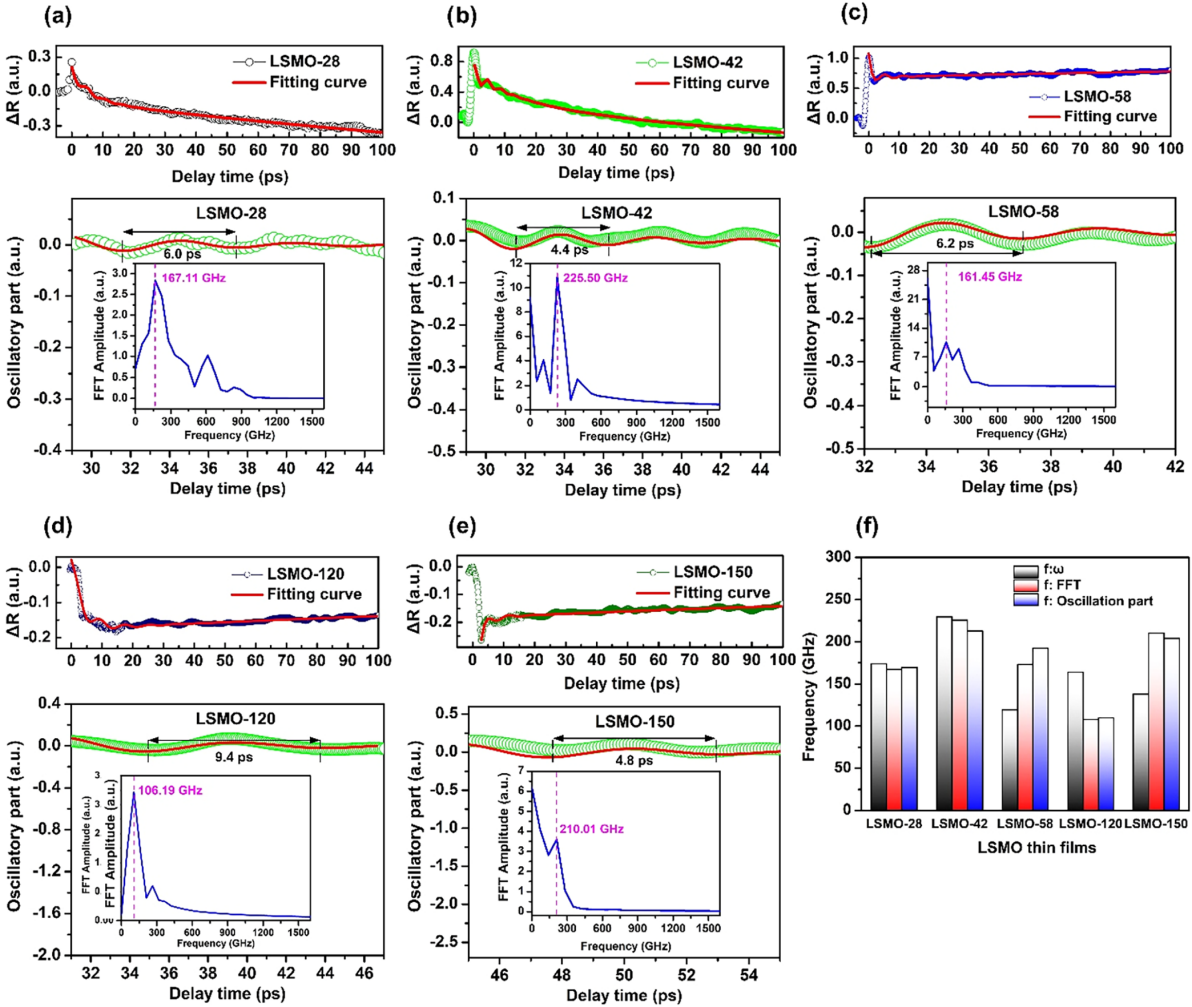
(a–e) Transient reflectivity changes (Δ*R*) and exponential fitting curves of LSMO thin films with thicknesses ranging from 28 to 150 nm, measured at room temperature. The oscillatory components are shown below, along with the insets displaying their corresponding Fast Fourier Transform (FFT) analyses. (f) Comparison of oscillation frequency calculations using different methods, including ω, FFT analysis, and extraction from the oscillation component.

**Table 2:** Dual Pump–Probe Fitting and FFT-Derived Parameters of LSMO Thin Films with Different Thicknesses^a^

	fitting results (eq 1)						FFT results	
samples	*T*_el‑la_ (ps)	*T*_sp‑la_ (ps)	*T*_mag_ (ps)	*T*_osc_ (ps)	ω (rad/ps)	*f* ^ω^ (GHz)	*f* ^FFT^ (GHz)	*T*_osc_^FFT^ (ps)
LSMO-28	261.12	5.99	247.77	6.04	1.09	173.93	167.11	5.98
LSMO-42	284.93	16.93	248.13	4.69	1.44	229.46	225.50	4.43
LSMO-58	274.14	0.90	300.68	5.63	1.03	164.71	161.45	6.19
LSMO-120	288.21	3.63	324.64	9.16	0.71	112.81	106.19	9.42
LSMO-150	294.75	0.47	337.52	4.73	0.87	138.03	210.01	4.76

a The oscillation period derived from FFT (*T*_osc_^FFT^ = 1/*f* ^FFT^) is included for comparison.

The results indicate a thickness-dependent variation in the strain oscillation frequency, with FFT-derived values of 167.11, 225.50, 161.45, 106.19, and 210.01 GHz for LSMO-28, LSMO-42, LSMO-58, LSMO-120, and LSMO-150, respectively (see insets for details). This trend suggests stronger lattice dynamics and faster relaxation processes as the thickness increases. The extracted oscillation decay times for these films indicate that thinner films exhibit slightly shorter decay times, which may be linked to enhanced strain interactions in thinner layers. Figure 5f compares the oscillation frequencies obtained through three methods: calculated from ω, FFT, and extracted from the oscillatory component. The results indicate consistent trends across the three methods, with slight variations in the calculated values. The general increase in frequency with film thickness up to 42 nm, followed by a decrease in thicker films, underscores the influence of film thickness on the strain-pulse dynamics and lattice interactions in LSMO thin films. These findings highlight the complex interplay between film thickness, lattice strain, and oscillatory behavior in determining the dynamic properties of the thin film.

Table 2 reveals several important trends regarding the dynamics of LSMO samples across different thicknesses. The electron–lattice relaxation time *T*_el‑la_ shows a gradual increase from 261.12 ps in the thinnest sample (LSMO-28) to 294.75 ps in the thickest sample (LSMO-150). This trend suggests that electron–lattice interactions slow down as the sample thickness increases, potentially indicating weaker electron–phonon coupling or structural changes in thicker films. The spin–lattice relaxation time *T*_sp‑la_ exhibits significant variability, starting relatively high at 5.99 ps in LSMO-28, peaking at 16.93 ps in LSMO-42, and then sharply decreasing for thicker samples, such as LSMO-58 and LSMO-150. This pattern implies enhanced spin–lattice coupling efficiency in intermediate and thicker films, which could be influenced by changes in magnetic domain dynamics. The magnetic order recovery time *T*_mag_ increases with thickness, ranging from 247.77 ps in LSMO-28 to 337.52 ps in LSMO-150. This observation indicates that thicker samples take longer to restore magnetic order after a disturbance, potentially due to increased complexity in their magnetic structures lead to increasing magnetic properties. The strain-pulse oscillation amplitude decay time *T*_osc_ varies, with a peak of 9.16 ps in LSMO-120, while thinner and thicker films show shorter decay times. This behavior suggests a resonance effect in intermediate thicknesses, where strain energy dissipation is more optimized. Finally, the oscillation frequency calculated by ω (*f*:ω) and its corresponding calculations from Fast Fourier Transform (*f*:FFT) show nonlinear trends. Both *f*:ω and *f*:FFT peak in thinner samples, particularly LSMO-42, indicate higher oscillation frequencies at this thickness, while frequencies derived from FFT show greater variability across the samples.

This nonlinear behavior can be understood in terms of strain-dependent acoustic phonon dynamics. In thin LSMO films grown on STO (lattice mismatch ∼1.28%), the film remains coherently strained at low thickness, leading to enhanced lattice stiffness and increased longitudinal sound velocity (*C*_a_). Based on the framework of coherent acoustic phonon generation in thin films established by Thomsen et al.^32^ and Ruello and Gusev,^33^ equidistant acoustic echoes are detected at arrival times *t* = 2*H*/*C*_a_, from which the oscillation frequency scales as *f* = *C*_a_/2*H*, where *H* is the film thickness. Accordingly, the relatively high frequency observed at 42 nm can be attributed to a maximized effective sound velocity under near-coherent strain conditions. As the film thickness increases beyond this regime, strain relaxation via misfit dislocations reduces the elastic stiffness and lowers *C*_a_, leading to a decrease in oscillation frequency. In thicker films, partial strain relaxation and structural inhomogeneity may also introduce multimode phonon contributions, resulting in the observed fluctuations in the FFT spectra. These findings collectively highlight the thickness-dependent interplay between electron–lattice, spin–lattice, and strain dynamics, suggesting that intermediate thicknesses may optimize certain dynamical properties, while thicker samples are characterized by slower relaxation and recovery processes.

The observation of large polarons with significantly faster electron–lattice dynamics in LSMO/BFO heterostructures provides clear advantages for energy-conversion applications. In standard LSMO, charge transport is dominated by Jahn–Teller small polarons, which exhibit strong localization and slow coupling times exceeding 200 ps. In contrast, the large polarons in LSMO/BFO, characterized by ultrafast dynamics down to 0.65 ps and lower-frequency oscillations (∼19–27 GHz), are more spatially delocalized, consistent with large-polaron behavior,^27,36^ and enhanced carrier mobility within the Fröhlich-type electron–phonon coupling framework.^28^ This faster carrier dynamics is expected to improve electrical conductivity and charge-transport efficiency, thereby reducing resistive losses and enhancing overall energy-conversion performance in oxide-based thermoelectric and photoelectric devices.

For magnetocaloric applications, the 120 nm single-layer LSMO film exhibits a *T*_C_ of ∼345 K, while a BFO overlayer tunes TC from 343 to 295 K via strain-mediated suppression of ferromagnetic exchange interactions,^37^ enabling the alignment of the magnetic entropy change peak with near-room-temperature operation.^38^ Furthermore, unlike current-driven switching in standard LSMO, the multiferroic BFO layer introduces strain-mediated magnetoelectric coupling,^9^ allowing electric-field control of exchange bias and enabling low-power spintronic switching without Joule-heating losses.

## 4 Conclusion

In summary, we investigated the influence of BFO thickness on the structural, magnetic, and carrier–lattice dynamics of LSMO/BFO heterostructures. Increasing the BFO thickness modifies the strain state of the LSMO layer, leading to a reduction in magnetization and a decrease in the Curie temperature from 343 to 295 K. A corresponding reduction in the coercive field is also observed, indicating that the magnetic properties of LSMO are strongly influenced by strain relaxation and interfacial coupling effects.

Time-resolved pump–probe spectroscopy reveals significant changes in carrier–lattice dynamics as the BFO thickness increases. The electron–lattice relaxation time decreases from 10.07 to 0.65 ps, accompanied by oscillatory strain responses with frequencies in the range of ∼19–24 GHz. These features indicate a transition toward large-polaron behavior in LSMO/BFO heterostructures, associated with enhanced electron–lattice coupling and reduced magnetic ordering. In contrast, single-layer LSMO films exhibit much slower relaxation dynamics (*T*_el–la_ > 200 ps) and higher oscillation frequencies (108–229 GHz), consistent with small-polaron dominated transport.

Overall, the results demonstrate that the strain environment introduced by the BFO layer provides an effective route for tuning magnetic properties and polaron dynamics in LSMO-based heterostructures. This strain-controlled modulation of carrier–lattice interactions offers insights into charge-transport mechanisms in complex oxide heterostructures, supporting energy conversion and spintronic applications by enabling improved carrier transport efficiency and tunable, low-power magnetic functionalities.

## Data Availability

Data will be made available on request.
